# The use of percutaneous coronary intervention in black and white veterans with acute myocardial infarction

**DOI:** 10.1186/1472-6963-6-107

**Published:** 2006-08-21

**Authors:** Charles Maynard, Haili Sun, Elliott Lowy, Anne E Sales, Stephan D Fihn

**Affiliations:** 1 Department of Veterans Affairs Puget Sound Health Care System, Health Services Research and Development, Seattle, Washington, USA

## Abstract

**Background:**

It is uncertain whether black white differences in the use of percutaneous coronary intervention (PCI) persist in the era of drug eluting stents. The purpose of this study is to determine if black veterans with acute myocardial infarction (AMI) are less likely to receive PCI than their white counterparts.

**Methods:**

This study included 680 black and 3529 white veterans who were admitted to Veterans Health Administration (VHA) medical centers between July 2003 and August 2004. Information for this study was collected as part of the VHA External Peer Review Program for quality monitoring and improvement for a variety of medical conditions and procedures, including AMI. In addition, Department of Veterans Affairs workload files were used to determine PCI utilization after hospital discharge. Standard statistical methods including the Chi-square, 2 sample t-test, and logistic regression with a cluster correction for medical center were used to assess the association between race and the use of PCI ≤ 30 days from admission.

**Results:**

Black patients were younger, more often had diabetes mellitus, renal disease, or dementia and less often had lipid disorders, previous coronary artery bypass surgery, or chronic obstructive pulmonary disease than their white counterparts. Equal proportions of blacks and whites underwent cardiac catheterization ≤ 30 days after admission, but the former were less likely to undergo PCI (32% vs. 40%, p < 0.0001). This difference persisted after multivariate adjustment, although measures of the extent of coronary artery disease were not available.

**Conclusion:**

Given the equivalent use of cardiac catheterization, it is possible that less extensive or minimal coronary artery disease in black patients could account for the observed difference.

## Background

In the past, black veterans hospitalized with acute myocardial infarction (AMI) were less likely to undergo cardiac revascularization procedures, including percutaneous coronary intervention (PCI), than their white counterparts [1-7]. These differences in the use of cardiac revascularization procedures were observed over 10 years ago in a health care system where there was and still is equal access to care. It is uncertain whether these differences in the use of PCI persist in the era of drug eluting stents. It is possible that black patients are less likely to be the beneficiaries of new technology due to receiving care at hospitals with lower procedure rates and greater disparity in racial composition [8]. On the other hand, the indications for PCI in the setting of AMI have expanded significantly due to new technology, and it is possible that this trend has affected the proportions of black patients undergoing PCI. The purpose of this study is to determine if in the era of drug eluting stents, black veterans with AMI are less likely to receive PCI than their white counterparts.

## Methods

### Patient population

Between July 20, 2003 and August 19, 2004, there were 6262 veterans who were admitted to Veterans Health Administration (VHA) medical centers and developed AMI that was documented by standard electrocardiographic, troponin, and/or other clinical evidence. Not included in this count were patients who were transferred into VHA hospitals from other medical facilities, because important baseline information was not available. Also excluded were patients who had AMI while hospitalized for another medical condition.

Information for this study was collected as part of the VHA External Peer Review Program for quality monitoring and improvement for a variety of medical conditions and procedures, including AMI. Patients with International Classification of Diseases 9^th ^Revision diagnosis codes 410.xx were identified from administrative data housed at the Austin Automation Center. Working with the peer review abstraction contractor, West Virginia Medical Institute, the VHA Office of Quality and Performance generated a list of patients that was transmitted to VHA facilities where both paper and electronic medical records were manually abstracted by trained abstractors using standard reporting forms. Abstracted data were then entered into a database maintained by the contractor.

### Study variables

We used information from the peer review database and Department of Veterans Affairs workload files to describe demographics and personal characteristics, vital status and cardiac procedure use after hospital discharge [9]. Race/ethnicity was obtained from the medical inpatient datasets and outpatient care files at Austin and was classified as 1) Hispanic, 2) Black, 3) White, 4) Other, or 5) Unknown.

Medical centers were classified according to type of invasive cardiac procedures offered: 1) no cardiac catheterization, 2) cardiac catheterization only, 3) PCI, or 4) full range of procedures including coronary artery bypass graft surgery. Clinical information such as cardiac medications, time from symptom onset to hospital admission, initial heart rate, systolic and diastolic blood pressures, and initial symptoms were obtained from the medical record. The initial electrocardiographic diagnosis of ST elevation myocardial infarction included those with ST elevation ≥ 1 millivolt in 2 or more contiguous leads and/or left bundle branch block. The remainder of patients had non-ST elevation AMI. Both the initial and highest troponin values were recorded; positive tests were determined according to criteria for the particular type of assay used. In both groups, over 99% of AMIs were confirmed by troponin elevations.

Cardiac treatments and procedures were recorded as part of chart abstraction, but workload data from the Austin Automation Center were also accessed to determine cardiac procedure use after discharge. For patients who presented to the hospital with ST segment elevation, PCI within 12 hours of admission was defined. In addition, 30-day rates of cardiac catheterization, PCI, and coronary artery bypass surgery were constructed.

### Statistical methods

Comparisons between black and white patients were made with the Chi-square test for categorical variables and the 2 sample t-test for continuous measures. Logistic regression was used to further examine the association between race and the use of PCI performed ≤ 30 days after admission. We used backward stepwise logistic regression to select predictors of PCI use. After statistically significant predictors were selected from candidate variables in tables 1 and 2, the black/white variable was forced in to determine its association with PCI use. Analyses were run with and without a cluster correction for medical center.

### Ethics and consent

This study was approved for expedited review by the University of Washington Institutional Review Board and was granted a waiver of informed consent.

## Results

From the 6262 individuals in this study, only black (n = 680) or white (n = 3529) patients were included, resulting in 4209 individuals. Excluded were those of other racial or ethnic groups (n = 388) or those whose race was unknown (n = 1665). Race/ethnicity was unknown for over 25% of individuals. Compared to their white counterparts, black individuals, who comprised 16% of the study group, were younger, less often had lipid disorders, previous coronary artery bypass graft surgery, or chronic obstructive pulmonary disease but more often had diabetes, renal disease, or dementia (table 1). In addition, black patients lived closer to VHA medical centers and more often resided in the South region of the United States. Significantly, a higher proportion of black veterans smoked in the past year. The distribution of cardiac medications used prior to admission was similar in the 2 groups.

With respect to presenting signs and symptoms, black and white veterans were similar, except that the former group had higher systolic and diastolic blood pressures on admission (table 2). Symptoms were similar in the 2 groups, although black individuals reported less shoulder pain. ST segment elevation on the initial electrocardiogram was present in 16% and 18% (p = 0.10) of black and white patients, respectively, although the first troponin assay was more often elevated in black veterans (76% vs. 69%, p = 0.001). The highest troponin reading was elevated in 99% of both groups.

There were distinct differences concerning the type of admitting hospital as well as the kind of cardiology services received. Almost 75% of black patients were admitted to VHA medical centers with capacity to perform PCI, compared to only 54% of white veterans. Significantly, 29% of white veterans were admitted to VHA hospitals without cardiac catheterization laboratories. Consistent with this result was the finding that white individuals were more likely to be transferred for PCI than were black patients (table 3). With respect to cardiology services, over 90% of veterans received them, although 49% of black individuals had a cardiology attending as compared to 38% of white veterans (p < 0.0001).

Rates of cardiac catheterization performed ≤ 30 days after admission were similar in the 2 groups as were rates of PCI performed ≤ 12 hours from admission (table 3). However, a significantly lower proportion of black veterans received PCI ≤ 30 days after admission (odds ratio = 0.71, 95% confidence interval = 0.60–0.85). Even among those who underwent cardiac catheterization, the rate of PCI was still lower in black veterans (48% vs. 59%, p < 0.0001). After adjusting for history of cerebrovascular disease, renal disease, chronic obstructive pulmonary disease, dementia, chest pain on admission, ST elevation on the admission electrocardiogram, prolonged chest pain, age, geographic region, and availability of PCI services at the admitting hospital, black patients had a 21% lower likelihood of undergoing PCI (odds ratio = 0.79, 95% confidence interval = 0.71–0.88). The confidence intervals were adjusted for the clustering of patients within medical centers and were not appreciably different from the unadjusted ones. The model had adequate discrimination and calibration as indicated by the area under the receiver operating characteristic curve (c statistic = 0.75, 95% confidence interval = 0.73–0.76) and statistically non-significant Hosmer-Lemeshow test (p = 0.50).

## Discussion

For many years, race and gender differences in the use of cardiac revascularization procedures have been noted, and it appears that they have persisted through time [10]. Similar differences have been noted in VHA, although gender differences are less relevant given that relatively few women with AMI are treated in VHA medical centers [1-7]. In the current study, a higher proportion of black veterans were younger, had diabetes mellitus, renal disease, dementia, or elevated blood pressure, although more white patients had lipid disorders, previous coronary artery bypass surgery, or chronic obstructive pulmonary disease. Significantly, a higher proportion of black individuals smoked cigarettes in the year prior to admission.

Even though they were more likely to be admitted to medical centers with interventional capability, black veterans were less likely to undergo PCI. This was true in both unadjusted and adjusted analyses. White patients were 2 times more likely to be transferred for PCI, and this increased the numbers undergoing PCI. Equal proportions of African-Americans underwent emergent or urgent PCI, and although white veterans more often underwent bypass surgery, the absolute difference was slightly less than 4%.

In this study there were no black-white differences regarding the use of cardiac catheterization, although this was not the case in a previous study conducted in VHA medical centers [11]. The fact that there were no black-white differences with respect to use of cardiac catheterization may provide a possible explanation for the observed difference with respect to PCI. If results of cardiac catheterization indicated that black veterans had minimal coronary artery disease or had lesions that were not amenable to intervention, then the difference could be explained. Unfortunately, measures of extent of coronary disease were not collected as part of the records abstraction. Peniston et al. compared the use of cardiac revascularization procedures in African-American and white veterans who underwent cardiac catheterization, and did not find racial differences after adjusting for extent of disease [7]. Whittle et al. reported that among clinically similar black and white veterans undergoing coronary angiography, black patients were less likely to have obstructive coronary disease [12].

In addition to the absence of catheterization results, another limitation of this study was that an undetermined number of veterans may have received PCI in non VHA hospitals. With the exception of Medicare data (which were not available for this study), these procedures are not recorded in national databases, and therefore could not be identified. If a higher proportion of white patients underwent PCI in non VHA hospitals, then the racial difference reported in this study may have been underestimated.

Over 25% of veterans were of unknown race. Recently the means for acquisition of race/ethnicity have changed in VHA. Race/ethnicity is now reported by the patient rather than determined by the individual provider or administrative assistant responsible for entering data into the electronic medical record. If the question is not asked or if the patient is not present when data are entered, then race/ethnicity is unknown.

Among those with unknown race, 43% had PCI within 30 days of admission; these individuals were on average 66 years of age and were less likely to have chronic conditions such as diabetes, congestive heart failure, lung disease, or cerebrovascular disease. Therefore patients with unknown race were relatively healthier and underwent PCI more often than their counterparts with known race. The problem of unknown race in VHA has been more apparent in the past several years. Therefore, for younger patients who did not use VHA health care in the 5 to 10 years prior to their events, race information from that time was not available to fill in missing information for 2003–2004. We were reluctant to impute race given the lack of adequate measures of characteristics highly correlated with race, such as income and education.

## Conclusion

In this era of drug eluting stents and more aggressive treatment for AMI, black-white differences in the use of PCI persist even in the VHA, where access to procedures is not based on the ability to pay. However, given the equivalent use of cardiac catheterization as well as the equivalent use of PCI within 12 hours of admission, it is possible that less extensive or minimal coronary artery disease in black veterans could explain the observed difference.

## Competing interests

The author(s) declare that they have no competing interests.

## Authors' contributions

CM-Conceived and designed study, performed data analysis, drafted manuscript.

EL-Acquired data, constructed study database, assisted with data analysis, revised manuscript critically for important intellectual content.

HS-Acquired data, constructed study database, assisted with data analysis, revised manuscript critically for important intellectual content.

AES-Revised manuscript critically for important intellectual content.

SDF-Revised manuscript critically for important intellectual content.

All authors have read and approved the final version of the paper.

## Pre-publication history

The pre-publication history for this paper can be accessed here:



## Figures and Tables

**Table 1:** Baseline demographics, medical histories, and prior cardiac medications by race

**Variable**	**Black (n = 680)**	**White (n = 3529)**	**P-value**
Mean age (years)	67 ± 13	70 ± 11	<0.0001
Age			<0.0001
< 50	9%	3%	
50–59	27%	18%	
60–64	9%	11%	
65–69	8%	11%	
70–79	26%	32%	
80–89	18%	22%	
90+	2%	2%	
Men	99%	98%	0.08
Body mass index	27.0 ± 6.0	28.0 ± 6.0	<0.0001
Miles from home to hospital	22 ± 88	41 ± 116	<0.0001
Geographic region of medical center			<0.0001
Northeast	16%	15%	
South	52%	43%	
Midwest	21%	21%	
West	11%	20%	
*Medical histories*			
Myocardial infarction	25%	28%	0.14
Lipid disorder	55%	64%	<0.0001
Coronary angioplasty	12%	14%	0.14
Coronary artery bypass surgery	10%	24%	<0.0001
Congestive heart failure	33%	31%	0.43
Diabetes mellitus	28%	23%	0.004
Renal disease	18%	13%	<0.0001
Cerebrovascular disease	9%	8%	0.46
Chronic obstructive pulmonary disease	13%	23%	<0.0001
Dementia	19%	15%	0.016
Cancer	10%	10%	0.81
Cigarette smoking in past year	41%	31%	<0.0001
*Previous medications*			
Aspirin	48%	49%	0.52
Beta blockers	51%	54%	0.13
Angiotensin converting enzyme inhibitors	50%	47%	0.24
Lipid lowering agents	48%	53%	0.031
Insulin	13%	14%	0.61
Platelet inhibitors	11%	12%	0.20

AMI Acute myocardial infarction

**Table 2:** Presenting signs and symptoms

**Variable**	**Black (n = 680)**	**White (n = 3529)**	**P-value**
Time from symptom onset to hospital admission			
≤ 1 hour	5%	6%	0.12
1–2 hours	9%	10%	
2–6 hours	20%	24%	
6–12 hours	13%	13%	
12–24 hours	17%	13%	
> 24 hours	36%	34%	
Heart rate	87 ± 25	87 ± 23	0.75
Systolic blood pressure	143 ± 33	138 ± 31	<0.0001
Diastolic blood pressure	80 ± 20	75 ± 19	<0.0001
Symptoms			
Chest pain	56%	58%	0.39
Pressure	17%	18%	0.58
Belching	6%	4%	0.23
Shoulder pain	21%	30%	<0.0001
Nausea	22%	18%	0.031
Diaphoresis	14%	17%	0.09
Dyspnea	45%	43%	0.36

**Table 3:** Treatments, transfer status, in-hospital events, and mortality

***Variable***	***Black (n = 680)***	***White (n = 3529)***	***P-value***
Cardiac catheterization ≤ 30 days from admission	59%	57%	0.52
Percutaneous coronary intervention ≤ 12 hours from admission	8%	8%	0.80
Percutaneous coronary intervention ≤ 30 days from admission	32%	40%	<0.0001
Drug eluting stent	7%	10%	0.024
Transfer to another hospital for percutaneous coronary intervention	8%	15%	<0.0001
Coronary artery bypass graft surgery ≤ 30 days from admission	5%	8%	0.001

AMI Acute myocardial infarction
